# Binary Logistic Regression Analysis of Foramen Magnum Dimensions for Sex Determination

**DOI:** 10.1155/2015/459428

**Published:** 2015-08-05

**Authors:** Venkatesh Gokuldas Kamath, Muhammed Asif, Radhakrishna Shetty, Ramakrishna Avadhani

**Affiliations:** ^1^Yenepoya Medical College, Nithyananda Nagar, Deralakatte, Mangalore, Karnataka 575018, India; ^2^Kanachur Medical College, Mangalore-Thokkottu-Konaje University Road, Kotekar, Natekal, Karnataka 575022, India

## Abstract

*Purpose*. The structural integrity of foramen magnum is usually preserved in fire accidents and explosions due to its resistant nature and secluded anatomical position and this study attempts to determine its sexing potential. *Methods*. The sagittal and transverse diameters and area of foramen magnum of seventy-two skulls (41 male and 31 female) from south Indian population were measured. The analysis was done using Student's *t*-test, linear correlation, histogram, Q-Q plot, and Binary Logistic Regression (BLR) to obtain a model for sex determination. The predicted probabilities of BLR were analysed using Receiver Operating Characteristic (ROC) curve. *Result*. BLR analysis and ROC curve revealed that the predictability of the dimensions in sexing the crania was 69.6% for sagittal diameter, 66.4% for transverse diameter, and 70.3% for area of foramen. *Conclusion*. The sexual dimorphism of foramen magnum dimensions is established. However, due to considerable overlapping of male and female values, it is unwise to singularly rely on the foramen measurements. However, considering the high sex predictability percentage of its dimensions in the present study and the studies preceding it, the foramen measurements can be used to supplement other sexing evidence available so as to precisely ascertain the sex of the skeleton.

## 1. Introduction

In a scenario with minimum forensic evidence, the identification of sex and ethnicity is a challenge and sex markers that are both accurate and reliable are an asset in investigation. Sex identification is a preliminary step in forensic analysis of skeletal remains and several researchers have attempted to analyse the sex predicting attributes of various parts of the crania. In a study conducted by Rogers in 2005, 17 morphological features of the skull were studied to determine the sex of the individual. Nasal aperture, zygomatic extension, malar size/rugosity, and supraorbital ridge were given primary significance followed secondarily by chin form and nuchal crest; mastoid size was found to be of tertiary significance followed by nasal size, mandibular symphysis/ramus size, forehead shape, palate size/shape, and other features [1]. It is therefore obvious that more than one parameter is essential in conclusively confirming sex and the more the parameters, the more the accuracy. In this context, every study that attempts to diagnose new sexing parameters adds to the accuracy of the forensic report. The osteometric analysis of skull base is of substantial significance due to the resistant nature of its parts such as the mastoid, foramen magnum, and the occipital condyles in explosions, fire trauma, and aircraft accidents. The base of the skull also has a favourable anatomical position as it is covered by the soft tissue and skeleton of the head that protects it from direct impact thus preserving this area for forensic testing. Holland in 1989 conducted a simulation study subjecting the skull base to temperatures equivalent to house fire accidents to observe for shrinkage in dimensions and noted that the accuracy of dimensions was not significantly altered and could still be used for sex determination [2]. The osteometric data and scientific literature available in this region are albeit limited considering their medico legal significance. “Variation in Size and in Symmetry of the Foramina of the Human Skull” by Berge and Bergman [3] and “Evaluation of the Foramen Magnum Dimension” by Sendemir et al. [4] are two studies on dimensions of the foramen. “Relation between Intracranial Volume and the Surface Area of the Foramen Magnum” by Acer et al. [5] and “Variability of Human Foramen Magnum Size” by Gruber et al. are a few relative studies. Gruber et al. have studied the relation of foramen to femur length and have also tried to observe for the presence of a secular trend in the foramen dimensions considering the fact that brain size and skull size show a secular trend [6]. There are also studies that analyse the sex determining attributes of the foramen magnum similar to the present study by Teixeria [7], Günay and Altinkök [8], and Raghavendra Babu et al. [9]. “A Morphological Comparison of the Foramen Magnum of the Male Middle Kyushites with That of Other Ethnological Groups” by Nakashima in 1986 defines the ethnical variation in foramen dimensions [10]. There are similar studies in CT scan images as well by Murshed et al. [11], Uysal et al. [12], and Uthman et al. [13]. The foramen being a transition zone between the cerebrum and the spinal cord has innumerable clinical implications as reported in several pieces of literature such as “Unusual Subacute Diencephalic Edema Associated with a Trapped Fourth Ventricle: Resolution following Foramen Magnum Decompression” by Udayakumaran et al. [14] and “Acute Foramen Magnum Syndrome Caused by an Acquired Chiari Malformation after Lumbar Drainage of Cerebrospinal Fluid: Report of Three Cases” by Dagnew et al. [15]. We hope that this study augments the existing literature and provides valuable information for future studies focusing on this region and also contributes to forensic analysis.

## 2. Materials and Methods

The morphometric analysis of the foramen magnum was conducted in the Department of Anatomy, Yenepoya Medical College, Mangalore. The study involved 41 male skulls and 31 female skulls, with the sex confirmation done previously from departmental records. All the skulls belonged to adults of age above 18 years. The measurements were done using a sliding digital calliper (Lianying 0005) graduated to the last 0.01 mm.

The technique used for taking the morphometry is as follows.

**Figure 1:**
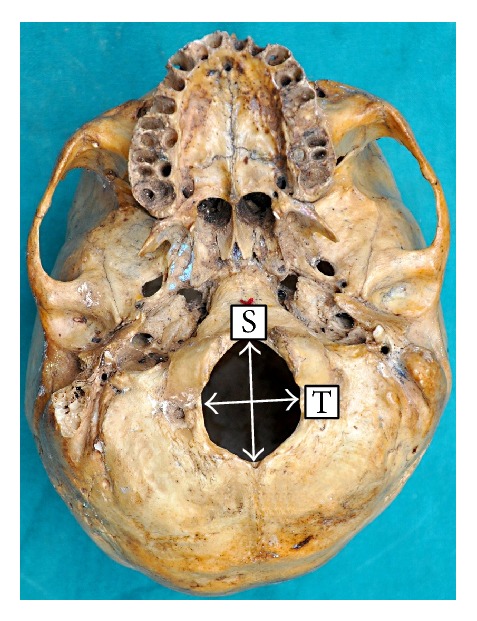
The base of the skull showing the sagittal dimension (S) and transverse dimension (T) of the foramen magnum.

The landmarks on the foramen are described in Figure 1.

(1) Technique used for measuring sagittal diameter is as follows.

The sagittal diameter (*s*) is the distance between Basion (B) and Opisthion (O). Basion and Opisthion are the points where the midsagittal plane intersects the anterior margin and the posterior margin of the foramen magnum, respectively.

(2) Technique used for measuring transverse diameter is as follows.

The transverse diameter (*t*) is the distance between the lateral margins of foramen magnum at the point of maximum lateral curvature.

The technique involved repetition of the measurements twice and the average results were compared. If there was a difference of more than 0.1 mm, then a third measurement was taken in accordance with the technique recommended by Krag et al., 1988, for spinal morphometry [16].

(3) The area of foramen magnum (*A*) was calculated using the following formulas:

(a) Radinsky's formula: *A* = 1/4 × *π* × *t* × *s*.
(b) Teixeria's formula: *A* = *π* × {(*s* + *t*)/4}^2^.

Statistical analysis was performed using SPSS (Statistical Package for Social Sciences, version 20.0) computer software (SPSS, Inc., Chicago, IL, USA), two-tailed Student's *t*-test (*p* < 0.05), Quantile-Quantile plot, linear correlation, Binary Logistic Regression (BLR), and Receiver Operating Characteristic (ROC) curve. Binary Logistic Regression is applied to obtain a predicting equation (BLR model) that estimates the sex of the individual. An equation is obtained for each variable and on applying the equation to the variable value a predicted value is obtained. In this model, the cut-off value is 0.5 and hence if the predicted value is equal to or more than 0.5 it is considered male and if it is less than 0.5 it is considered to be female. The predicted probabilities of BLR were analysed using Receiver Operating Characteristic (ROC) curve. The ROC curve is a strong indicator of the models ability to distinguish two groups and the area under the curve is used to measure the strength of the equation. If the area is less than 0.5, it indicates that any observation is purely a matter of chance and a value close to 1 indicates that the equation strongly discriminates two groups. The results of the present study are also compared with the previously published studies on morphometry and ethnicity using a scatter plot [10, 17].

## 3. Results

It was observed that on an average the sagittal diameters (*s*) were greater than the transverse diameter (*t*) (*p* < 0.001) and by conventional criteria this difference is considered extremely statistically significant and is also consistent with the shape of the foramen. The mean sagittal diameter in the present study is 32.26 mm and transverse diameter is 26.29 mm. The average and standard deviation values are shown in Table 1. The mean sagittal diameter in males is 33.21 mm and in females it is 30.99 mm. The mean transverse diameter in males is 26.92 mm and in females it is 25.45 mm.

The sagittal diameter ranges from a minimum value of 24.41 mm to a maximum value of 42.87 mm. The transverse diameter ranges from a minimum value of 20.22 mm to a maximum value of 34.08 mm. The most frequent value for sagittal diameter is 34.30 mm and in transverse diameter 27.34 mm, 27.94 mm, and 28 mm are frequently repeated values.

**Figure 2:**
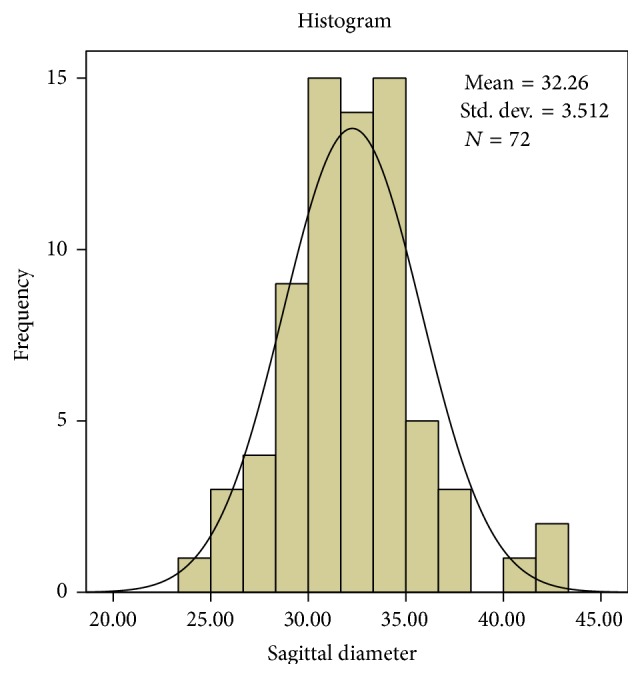
The values of sagittal diameter (mm) revealed a high interindividual variability.

**Figure 3:**
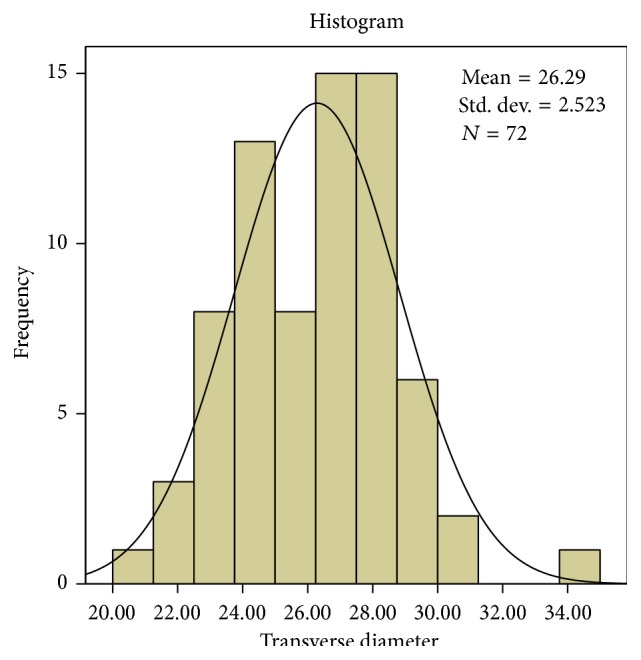
The values of transverse diameter (mm) revealed a high interindividual variability.

**Figure 4:**
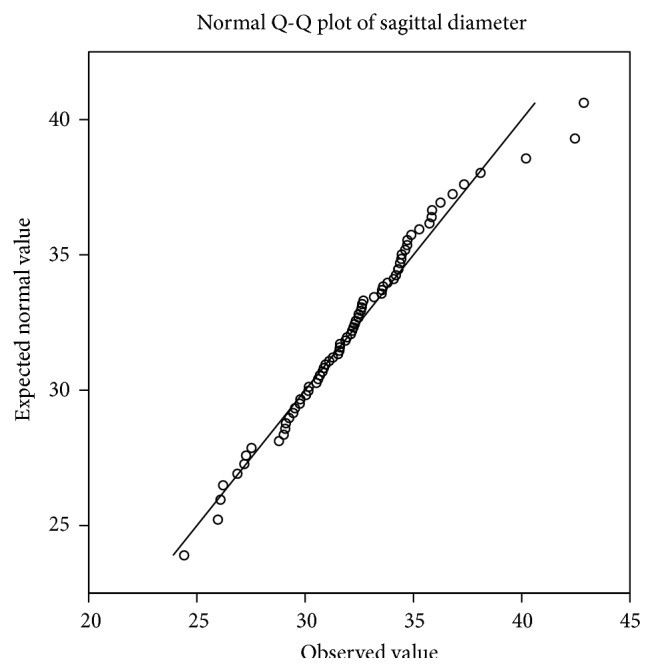
The Q-Q plot of sagittal diameter shows normal distribution.

**Figure 5:**
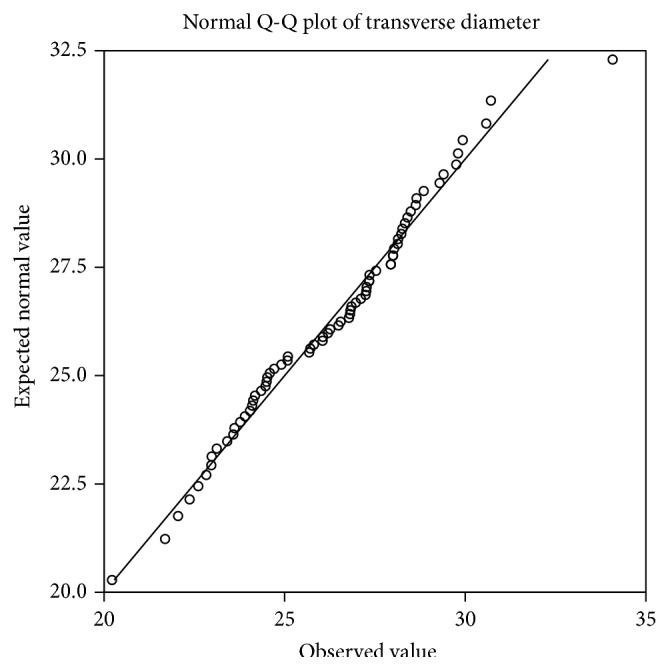
The Q-Q plot of transverse diameter shows normal distribution.

Interindividual variation is observed in both diameters and is shown in Figures 2 and 3. The Q-Q plot confirms normal distribution as shown in Figures 4 and 5.

**Figure 6:**
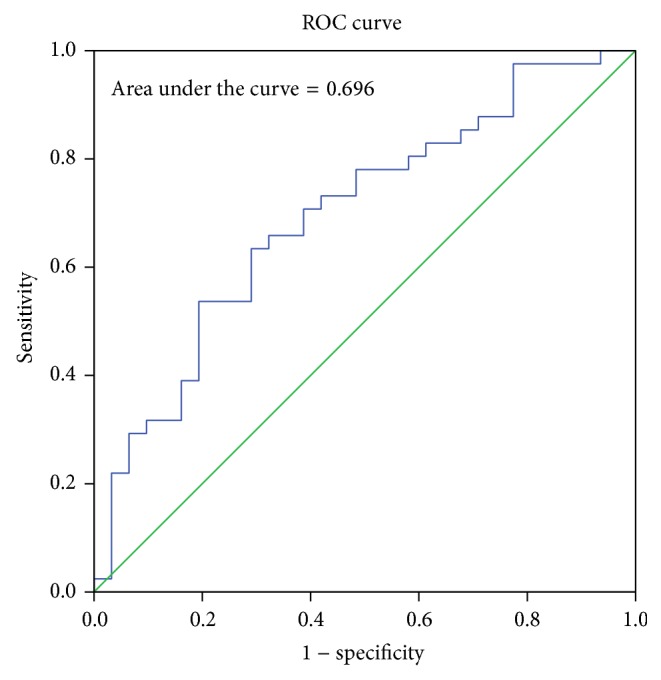
ROC curve for the predicted probabilities of sagittal diameter.

**Figure 7:**
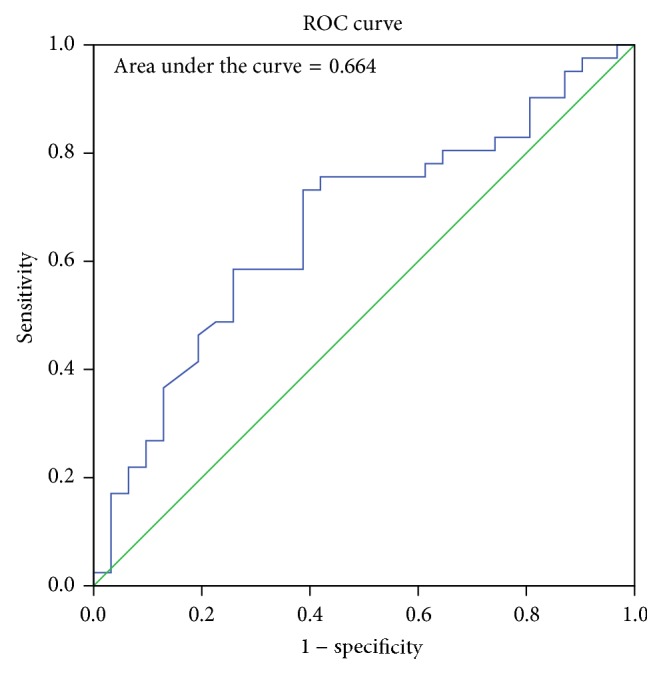
ROC curve for the predicted probabilities of transverse diameter.

**Figure 8:**
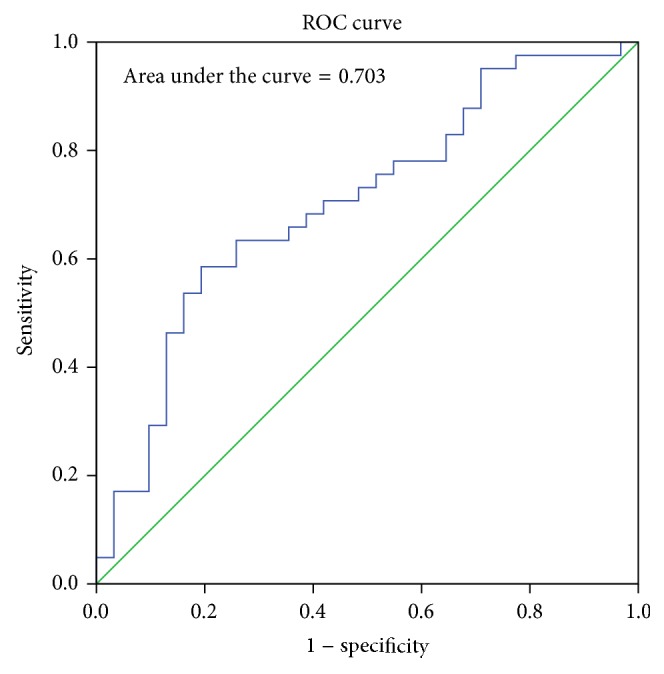
ROC curve for the predicted probabilities of area using Radinsky's formula.

**Figure 9:**
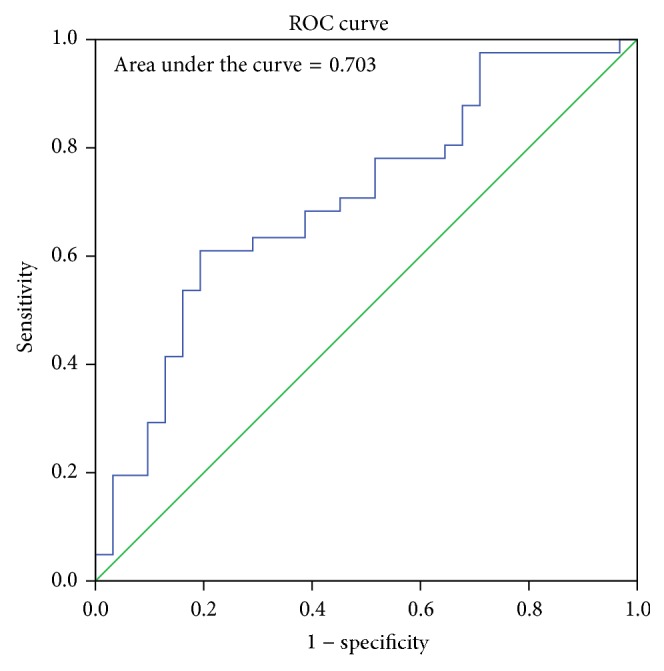
ROC curve for the predicted probabilities of area using Teixeria's formula.

The sex determining significance of both diameters and area of foramen was tested initially using Student's *t*-test and it was observed that the *p* value was significant for all the dimensions as shown in Table 2. The sagittal diameter analysis in both sexes revealed a *p* value of 0.007 and transverse diameter analysis revealed a *p* value of 0.014. The area of the foramen magnum measured using Radinsky's formula, Area (*R*), revealed a *p* value of 0.0034 and Teixeria's formula, Area (*T*), revealed a *p* value of 0.0036. All the values are <0.05 and hence statistically significant and the sex determining significance of the area of the foramen (*p* = 0.0034) and sagittal diameter (*p* = 0.007) is more than that of the transverse diameter (*p* = 0.014). The next step in analysis was to obtain an equation that determines the sex of the individual on applying the value of the variable. The BLR model for each variable is shown in Table 3 and on applying the model any predicted value <0.5 is considered to be female and equal to or more than 0.5 as male. The strength of each model was then tested by the area under the Receiver Operating Characteristic (ROC) curve drawn for the predicted probabilities of BLR. The ROC curve of each variable is shown in Figures 6, 7, 8, and 9. The area under the curve is a measure of the predictability of the variable in sexing the crania. The area is 0.696 for sagittal diameter, 0.664 for transverse diameter, and 0.703 for area of foramen measured by both methods. This suggests that the predictability of area is the highest with 70.3%, followed by sagittal diameter with 69.6%, and then the transverse diameter with 66.4% predictability. Also all the values of area under the curve are more than 0.5 which suggest that the variables significantly discriminate the two groups which in this case are males and females.

**Figure 10:**
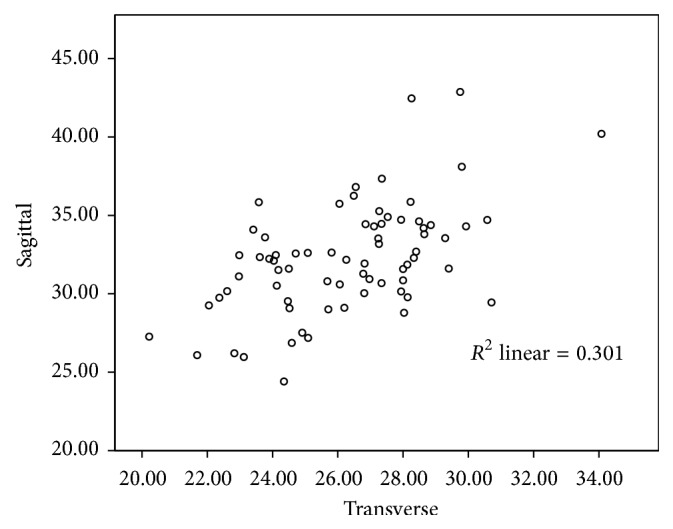
A moderately positive correlation between the sex-pooled sagittal and transverse diameter (mm) of the foramen magnum (*r* = 0.549, *p* < 0.001).

There is a moderately positive linear correlation between the sex-pooled sagittal and transverse diameters of the foramen (*r* value = 0.549) and the correlation is significant (*p* < 0.001) as shown in Figure 10.

**Figure 11:**
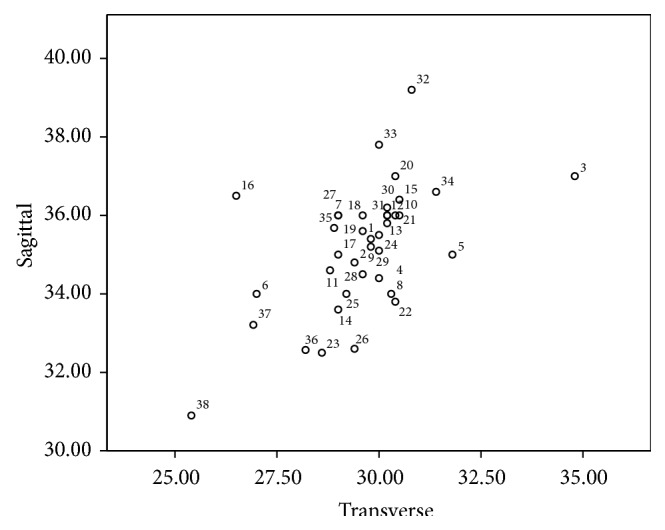
It depicts the ethnic variability of the foramen magnum dimensions in millimetres in different ethnic groups. Most of the values are obtained from a study by Martin, 1928 [17], and Nakashima, 1986 [10]. The data below shows the regions represented by the numbers. ^*∗*^A study by Gruber et al., 2009 [6]. ^*∗∗*^A study by Raghavendra Babu et al., 2012 [9]. The numbers 37 and 38 represent the present study in males and females, respectively. 1 = Germans m, 2 = Swiss f, 3 = Elsasee m, 4 = Elsasee f, 5 = Romans m, 6 = Romans f, 7 = Tyrolese, 8 = Bavarians m, 9 = Romans f, 10 = Swiss Wallis m, 11 = Swiss Wallis f, 12 = Swiss-Danis, 13 = Ainos m, 14 = Ainos f, 15 = Japanese m, 16 = Japanese f, 17 = Bashkirs m, 18 = Telengets, 19 = Chinese, 20 = Buryats, 21 = Torguts, 22 = Malays m, 23 = Malays f, 24 = Australians m, 25 = Australians f, 26 = Paltacalos m, 27 = Paltacalos f, 28 = Middle Kyushites m, 29 = Kantoites m, 30 = North Kyushites m, 31 = Yoron Islander/Fuschen Chinese, 32 = Kikai Islanders m, 33 = Shlingol Mongolians m, 34 = Central Western Europe^*∗*^, 35 = Indian male^*∗∗*^, 36 = Indian female^*∗∗*^, 37 = Indian male (present study), and 38 = Indian female (present study).

The ethnic variability of the foramen magnum dimensions is depicted in Figure 11 using a scatter plot. Most of the values are obtained from a study by Martin, 1928 [17], and Nakashima, 1986 [10]. The values in Central Western Europe are obtained from a study by Gruber et al. [6] and those of India from a study by Raghavendra Babu et al. [9]. The dimensions of the foramen measured in this study represent the south Indian ethnic group.

## 4. Discussion

The morphometric variability observed in various studies is due to the diverse ethnic groups involved. Studies by Kanodia et al. in 2012 [18], Shepur et al. in 2014 [19], and Patel and Mehta in 2014 [20] are a few recent studies on morphometric variations of the foramen. The study by Kanodia et al. involved 100 normal computerized tomography scans of posterior cranial fossa and 100 dry adult skulls without any bony abnormality; that by Shepur et al. involved 150 dry skulls and 30 CT scan images and Patel and Mehta studied 100 dry adult skulls. In all the studies, the sagittal diameter was significantly larger than the transverse diameter and this is consistent with the shape of the foramen. In almost all the studies, the mean dimensions of the foramen were more in males than in females. This was observed by several authors such as Olivier [21], Routal et al. [22], Sayee et al. [23], Gruber et al. [6], and Raghavendra Babu et al. [9]. However, the significance of this observation in sex estimation varied depending on the ethnic group involved, size of the study sample, and the statistical analysis applied in the study. In the present study, the mean sagittal diameter is 33.21 mm in males and 30.99 mm in females and the mean transverse diameter is 26.92 mm in males and 25.45 mm in females. When compared to several other studies mentioned above, the mean values are 3 to 5 millimetres lower and this is likely to be due to the ethnic variation. The scatter plot in Figure 11 compares the sagittal and transverse diameters of several ethnic groups and their relation to the present study. The area estimation in this study was done using two formulas, Radinsky's and Teixeria's formulas. Although the values of area obtained by Teixeria's formula, Area (*T*), were more than that obtained by Radinsky's formula, Area (*R*), on statistical analysis by BLR and ROC, the area under the curve was observed to be 0.703 for both areas. This suggests that the predictability of the area is the same (70.3%) irrespective of the formula applied. In a study by Kanchan et al., in 2013, in 118 dry skulls in south Indian ethnic group, it was observed that the areas of the foramen calculated by Radinsky's and Teixeria's formulae are better predictors of sex than the sagittal and transverse dimensions as noted in our study. However, this study applies *t*-test to analyze the significance and does not analyze the predictability percentage and Binary Logistic Regression [24].

A literature defined conclusion of sexing accuracy of the foramen magnum is difficult because there is a group of studies that conclude that the foramen can help in sexing and there are also substantial studies that contradict this view. A study similar to the present one in Indian population by Raghavendra Babu et al. using Binary Logistic Regression and Receiver Operating Characteristic revealed a higher predictability of dimensions. The predictability was 86.5% for anteroposterior diameter, 65.4% for transverse diameter, 81.6% and 82.2% for area by Radinsky and Teixeria, respectively, and 88% predictability when anteroposterior and transverse diameters were combined in BLR. However, despite such a probability, the authors conclude that the sexing potential is limited due to considerable overlapping of male and female values [9]. The findings in our study are in accordance with this study and we also have a similar view. The area under the curve is above 0.5 which suggests that there is a relation between the dimensions and sex but this relation must be taken along with other evidence to confirm sex precisely. Gapert et al. have also done a similar study in British ethnic group using discriminant function and regression analysis and predicted a sexing accuracy of 70.3% [25]. Edwards et al. in 2013 analyzed the CT scans of 250 adults from Swiss ethnic group to determine the value of foramen magnum dimensions in sexing crania. Statistical analysis revealed 66% accuracy in cranial sexing by discriminant function analysis and Binary Logistic Regression showed an overall classification rate of 66.4%. The morphology of the foramen magnum was classified by visual assessment into seven shape types. This study concludes that while foramen magnum dimensions appear to demonstrate statistically significant differences between the sexes, isolated use of this method is not advisable unless as a suggestive finding when other features of assessment are absent or limited [26]. In a study by Singh and Talwar, in 2013, involving fifty adult skulls it was noted that the accuracy of sex prediction based on discriminant function analysis ranged from 66% to 70% and maximum bicondylar breadth was found to be more discriminating variable providing an accuracy of 66% [27].

Studies by Catalina-Herrera [28], Holland [2], Uysal et al. [12], and Uthman et al. [13] also conclude that the foramen exhibits sexual dimorphism. The study by Uysal et al. uses Fisher's linear discriminant function test on three-dimensional computed tomography measurements and concludes that 81% accuracy in sexing is possible with foramen width and right condyle dimensions [12]. In the study by Uthman et al., helical CT scanning is used and the foramen diameters, area, and circumference were statistically analyzed using discriminant analysis and multiple regression analysis. The circumference and area were the best discriminant parameters for sex determination with an overall accuracy of 67% and 69.3%, respectively [13]. This observation of 69.3% predictability for area is close to our study which also shows 70.3% predictability for area in sex determination. In a study by Ukoha et al. in 2011, in Nigerian ethnic group, involving 100 skulls, sectioning point derived by the discriminant function was used for sexing and it was concluded that foramen magnum dimensions exhibit sexual dimorphism [29]. The study by Jain et al. in 2013, in north Indian ethnic group involving 68 adult skulls, also confirms its sexing potential [30]. A study by Burdan et al., in 2012 in eastern European ethnic group, using 3D computer tomography images of 313 individuals, revealed significantly higher mean values of length, breadth, and area of foramen magnum in males than in females. A significantly positive correlation was also observed between length and breadth of foramen similar to present study [31]. A study by Shanthi and Lokanadham in 2013 in south Indian ethnic group in hundred skulls revealed an extremely significant *p* value less than 0.001 for sagittal diameter and a significant *p* value of 0.015 for transverse diameter [32]. In a study by Loyal et al. in 2013, in Kenyan ethnic group involving two hundred and two adult skulls, it was observed that the shape of the foramen magnum was oval, circular, and polygonal in 13%, 24%, and 63% of the cases, respectively. The study concluded that the shape of the foramen does not show sexual dimorphism and cannot be used to ascertain the gender of skulls [33]. There are also studies that contradict these views and deny the existence of sexual dimorphism in foramen dimensions. This includes studies by Eisenstein in 1977 [34], Porter et al. in 1978 [35], Hasue et al. in 1983 [36], Routal et al. in 1984 [22], Kikuchi et al. in 1984 [37], Sayee et al. in 1987 [23], Günay and Altinkök in 2000 [8], Deshmukh and Devershi in 2006 [38], and Gruber et al. in 2009 [6]. The study by Gruber et al. was aimed at identifying a secular trend in foramen dimensions and the sample with known sex was very small and hence a limitation in the study [6]. The studies by Routal et al. and Sayee et al. were based on identification points (IP) and demarking points (DP) analysis and that of Deshmukh and Devershi was based on univariate analysis [22, 23, 38]. In the study by Günay and Altinkök, sex estimation was based on the area of foramen and correlation coefficient analysis and the correlation coefficient was 0.27 and hence was insignificant [8]. A study by Cui and Zhang in 2013 in Chinese ethnic group involved 276 skulls, all male, and examined the relationship between the stature of individual and the dimensions of the foramen magnum. Measurements were used for stature reconstruction and statistical analyses indicated that bilateral variation is insignificant for all measurements except maximum length of condyle in the southern Chinese population with a *p* value less than 0.01 and that the northern and southern populations differ significantly only in the minimum distance between condyles. Linear and multiple regression equations for stature estimation were established in this study [39].

The present study shows the sex predictability of each dimension. It is observed that the sex predictability is highest for area (70.3%), followed by sagittal diameter (69.6%), and least for transverse diameter (66.4%). This observation is similar to that of Uthman et al. (69.3% for area) [13] and Raghavendra Babu et al. which also predicts a greater predictability for area (81.6%, 82.2%) and sagittal diameter (86.5%) compared to transverse diameter (65.4%) [9].

## 5. A Note on Basicranial Embryogenesis and Related Complications

Nemzek et al. in 2000 described the development of the basicranium using twenty-nine formalin fixed foetal specimens ranging from 9 to 24 weeks of gestation, which were examined using radiological imaging techniques. The ossification and embryogenesis of basicranium at different periods of gestation were thus assessed. It was observed that the skull base develops from three pairs of central cartilaginous precursors, with lateral cartilaginous centres contributing to completion of its formation, with the remaining components developing from membranous ossification. The central paired cartilages include the prechordal cartilages in front of the notochord which gives rise to sphenoid body anterior to tuberculum, chiasmatic sulcus, olivary eminence, and perpendicular plate of ethmoid bone and crista galli, the hypophyseal cartilages surrounding the pituitary which gives rise to sphenoid body posterior to tuberculum, sella turcica, dorsum sellae, and part of clivus, and the parachordal cartilages which contribute to rest of clivus, anterior and posterior occipital condyles, hypoglossal canal, and the jugular tubercle. The lateral contributions are by the lateral cartilages, namely, the orbitosphenoid, which forms lesser wing of sphenoid, anterior clinoids, and planum sphenoidale, the alisphenoid that forms the greater wing of sphenoid and the medial pterygoid plate. Some components such as the orbital plate of frontal bone, greater wing of the sphenoid, lateral pterygoid plate, part of squamous occipital bone, and palatine bones develop from membrane [40]. The occipital bone and the foramen magnum are formed from the union of four primary cartilaginous centres that encircle the medulla oblongata which include two lateral exoccipital segments on either side, the posterior squamous occipital bone and the anterior basiocciput [41].

The embryological defects observed in the basicranium are well described by Tokumaru et al. in their study “Skull Base and Calvarial Deformities: Association with Intracranial Changes in Craniofacial Syndromes.” The authors have stated that deformities of the skull base were commonly recognized in their patients and such anomalies were of considerable clinical importance as several vital structures pass through the basicranial foramina. It was observed that hypoplasia of basicranial foramina results in cranial neuropathies and moreover chondroplasia with a small skull base was a potential cause for hydrocephalus in several patients. Similarly, deformity of the optic canals and superior orbital fissures may compress the optic nerve or reduce ocular motility thus impairing vision [42]. A cerebellum located in a hypoplastic posterior cranial fossa can herniate either upwards or downwards through the foramen magnum. Crouzon syndrome and Apert syndrome are two clinical syndromes involving basicranium with high frequency of cleft palate, bifid uvula, and high arching palate [43]. Wang et al. in 2005 have conducted a study on pathogenesis of Apert syndrome and observed that the syndrome is characterised by midline sutural defects, craniosynostosis, abnormal osteoblastic proliferation and differentiation, and cartilaginous anomalies of the basicranium with defective development of brain and other viscera [44]. Reid et al. in 2015 conducted a comprehensive study on basicranial malformations in a holoprosencephalic foetus with trisomy 18 (Edwards' syndrome) with synophthalmic cyclopia and alobar holoprosencephaly. The genetic defect was a translocation at 18p11.31. The authors observed bilateral absence of the anterior cranial fossa and ethmoid bone and the middle cranial fossa was shifted anteriorly with the foramina either missing or displaced. The extensive basicranial malformations observed have been attributed to transcription factors such as TGIF located on chromosome 18 which plays a major role in synchronous development of neural structures (brain) and the supporting skeletal components (skull) [45].

## 6. Conclusion

The sexual dimorphism of foramen magnum dimensions is established in the study. However, due to considerable overlapping of male and female values, it is unwise to singularly rely on the foramen measurements. However, considering the high sex predictability percentage of their dimensions in the present study and the studies preceding it, the foramen measurements can be used to supplement other sexing evidence available so as to precisely ascertain the sex of the skeleton.

## Figures and Tables

**Table 1:** It depicts descriptive statistics of the sagittal and transverse diameters (mm).

	*N*	Mean	Standard deviation	Minimum–maximum
Sagittal diameter	72	32.26 mm	3.5	24.41–42.87
Transverse diameter	72	26.29 mm	2.5	20.22–34.08

**Table 2:** It depicts the descriptive analysis of sagittal and transverse diameters (mm) in both sexes.

	Male (*n* = 41)		Female (*n* = 31)		*p* value
	Range	Mean (S.D)	Range	Mean (S.D)	
Sagittal diameter	26.09–42.87	33.21 (3.25)	24.41–42.46	30.99 (3.49)	0.007
Transverse diameter	21.69–34.08	26.92 (2.52)	20.22–30.71	25.45 (2.31)	0.013
Area (*R*)	444.63–1076.44	705.97 (119.85)	433.24–942.79	622.64 (109)	0.0034
Area (*T*)	448.43–1083.80	715.32 (122.05)	443.01–982.40	630.57 (113.14)	0.0036

**Table 3:** It depicts the Binary Logistic Regression model for the estimation of sex from the foramen dimensions. (The cut-off value is 0.5.)

Variable	BLR model	Wald	*p* value
Sagittal diameter (*s*)	−6.628 + 0.216 (*s*)	6.409	0.011
Transverse diameter (*t*)	−6.406 + 0.255 (*t*)	5.614	0.018
Area (*R*)	−4.307 + 0.007 (*R*)	7.392	0.007
Area (*T*)	−4.246 + 0.007 (*T*)	7.251	0.007
